# Energetic Cost of *Ichthyophonus* Infection in Juvenile Pacific Herring (*Clupea pallasii*)

**DOI:** 10.1155/2011/926812

**Published:** 2011-03-28

**Authors:** Johanna J. Vollenweider, Jake L. Gregg, Ron A. Heintz, Paul K. Hershberger

**Affiliations:** ^1^Auke Bay Laboratories, Alaska Fisheries Science Center, National Marine Fisheries Service, National Oceanic and Atmospheric Administration, 17109 Point Lena Loop Road, Juneau, AL 99801, USA; ^2^Marrowstone Marine Field Station, and Western Fisheries Research Center, United States Geological Survey, 616 Marrowstone Point Road, Nordland, WA 98358-9633, USA

## Abstract

The energetic costs of fasting and *Ichthyophonus* infection were measured in juvenile Pacific herring (*Clupea pallasii*) in a lab setting at three temperatures. Infected herring incurred significant energetic costs, the magnitude of which depended on fish condition at the time of infection (fat versus lean). Herring that were fed continually and were in relatively good condition at the time of infection (fat) never stored lipid despite *ad libitum* feeding. In feeding herring, the energetic cost of infection was a 30% reduction in total energy content relative to controls 52 days post infection. Following food deprivation (lean condition), infection caused an initial delay in the compensatory response of herring. Thirty-one days after re-feeding, the energetic cost of infection in previously-fasted fish was a 32% reduction in total energy content relative to controls. Body composition of infected herring subsequently recovered to some degree, though infected herring never attained the same energy content as their continuously fed counterparts. Fifty-two days after re-feeding, the energetic cost of infection in previously-fasted fish was a 6% reduction in total energy content relative to controls. The greatest impacts of infection occurred in colder temperatures, suggesting *Ichthyophonus*-induced reductions in body condition may have greater consequences in the northern extent of herring's range, where juveniles use most of their energy reserves to survive their first winter.

## 1. Introduction

*Ichthyophonus* is a commonly occurring Mesomycetozoean parasite that has been reported in more than 100 species of fish [1–3]. *Ichthyophonus *is highly pathogenic to Pacific herring (*Clupea pallasii*) [4] and occurs in high prevalence and intensity in herring populations throughout the northeast Pacific Ocean [5–7]. Epizootics of the parasite are known to structure herring populations and populations of other fish species [1, 2, 8, 9]. 

Infection by *Ichthyophonus* is not 100% fatal (see Gregg et al. this issue). Infection elicits systemic inflammation and tissue destruction resulting in vital organ failure [10]. In survivors, quantification of reduced fitness or identification of chronic problems associated with the parasite is limited to a study which found decreased swimming performance in salmon (*Oncorhynchus*) as a result of tissue damage. Authors surmised that poor swimming ability may lead to fatigue and elevated depletion of lipid reserves that would ultimately interfere with migrations [11]. Differences in swimming stamina between *Ichthyophonus*-infected and uninfected cohorts are more pronounced at higher temperatures [12] where metabolic energy costs are higher. Increased energetic costs and exhaustion of lipid stores could have a host of additional consequences particularly in relation to other energetically demanding processes such as maturation, spawning, or overwinter survival.

At high latitudes, overwinter survival of young-of-the-year fish has been identified as a critical period determining year class strength of many fish species [13–15]. Nearer the poles, growing seasons are short which limits the time in which animals can attain energy. During the brief summer months, juvenile fish have high energetic demands with conflicting requirements. They must allocate energy to growth to increase foraging opportunities and avoid predation, as well as allocate energy to storage to supply endogenous energy during winter when prey is scarce [16]. Additional energetic demands, such as those imposed by parasite infection, could have serious consequences for fish already contending with energy limitations.

Pacific herring are a northern species adapted to the extreme seasonal conditions of the North Pacific. Herring occupy an intermediate trophic level, transferring energy from zooplankton to higher level consumers such as many fish species, marine mammals, and seabirds [17–19]. As zooplanktivores, they are tightly linked to seasonal fluctuations in productivity [20]. Consequently, their body composition fluctuates significantly in a seasonal manner. Energy content of age-0 herring increases throughout the summer and autumn as herring assimilate energy from foraging and subsequently declines over winter when prey availability is low and endogenous energy stores are utilized. In spring herring reach an energetically sensitive condition when their lipid and energy content are at a minimum [21, 22]. In this low-energy state, juvenile herring may be most susceptible to stress, including disease.

The objectives of the study were to measure the energetic condition of juvenile herring infected with *Ichthyophonus* (relative to control fish) under a variety of energetic states and temperatures to mimic various seasonal environmental scenarios found in the northern Gulf of Alaska. Specifically, we compared the body composition of laboratory-reared herring infected with *Ichthyophonus* to controls that were (1) relatively fat, having fed continuously; representing fish in the late summer and autumn, and (2) relatively lean, having undergone extensive fasting followed by a period of refeeding; representing herring in the spring that had undergone winter fasting and commenced foraging on spring plankton blooms. The latter tests were conducted at three temperatures in order to understand how the cost of infection in the most energetically sensitive fish may vary throughout their range (California to Alaska). 

## 2. Methods

### 2.1. Study Design

To ensure herring were free of *Ichthyophonus* and immunologically naïve, naturally spawned herring eggs were collected from the southern Strait of Georgia (48°55.85′ N, 122°48.15′ W) on 5 May 2008 and held in filtered, UV-irradiated seawater at the US Geological Survey Marrowstone Marine Field Station, in Nordland, Wash, USA (48°06.08′ N, 122°41.32′ W). Eggs hatched on 18 May 2008 and herring were cultured to age 1 according methods outlined by Gregg et al. (this issue).

**Figure 1:**
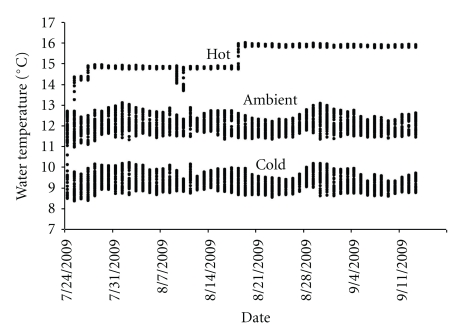
Experimental water temperatures over the course of the study. Ambient water from Puget Sound (mean = 12.0°C) was heated and chilled in head tanks to obtain the “hot” (mean = 15.0°C) and “cold” (mean = 9.5°C) treatments, respectively.

Herring were separated into two groups to simulate the impact of *Ichthyophonus* infection on fish in (1) autumn (relatively fat fish), and (2) spring (relatively lean fish) (Figure 1). “Autumn fish” were fed *ad libitum* and maintained in ambient water temperature for the duration of the experiment. On day 0, herring were divided into infected and uninfected (control) treatments. Infection with *Ichthyophonus* occurred via intraperitoneal injection according to procedures outlined by Gregg et al. (this issue). Control fish in the uninfected treatment were injected with sterile phosphate buffered saline to provide equivalent handling conditions. 

**Figure 2:**
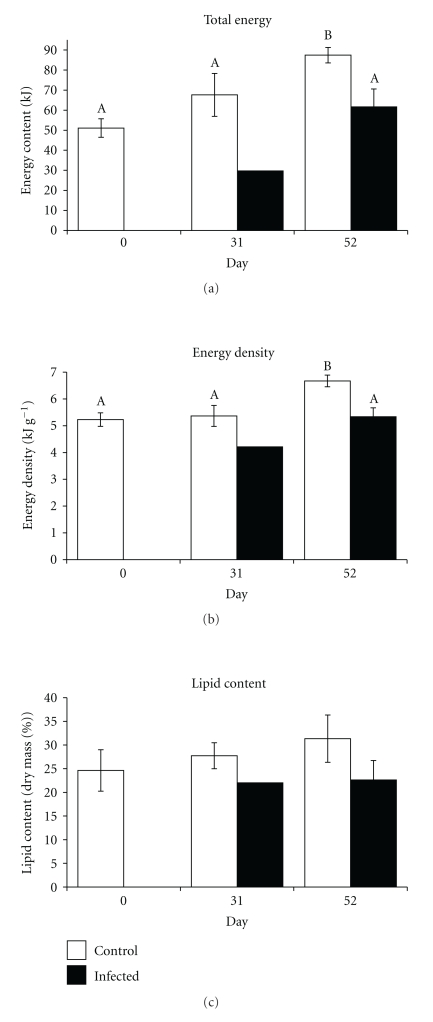
Energetic cost of *Ichthyophonus *infection in “autumn” young-of-the-year Pacific herring depicted by total energy content, energy density, and lipid content (% dry mass). Fish represented in this figure were cultured in ambient water temperature (9.5°C). Different letters represent statistical differentiation. Lack of letters indicates no statistical differentiation. Low sample size of infected fish on day 31 precludes statistical tests.

Prior to the study initiation, “spring herring” underwent a 56-day fasting period to simulate limited food availability in winter. On day 0, fish were also divided into infected and uninfected (control) treatments and were maintained in three water temperatures (cold, ambient, hot). At the time of infection, herring were randomly assigned to triplicate tanks for each treatment/temperature condition. After herring were distributed to experimental tanks, ambient seawater pumped from Puget Sound was gradually manipulated over 24 hours to provide cold and hot treatments. The temperature regulating system allowed all temperatures to vary with ambient seawater temperature while maintaining relatively consistent separation between them. Water temperature was recorded every 30 minutes (Figure 2). Mean water temperatures for the three temperature conditions were 9.5, 12.0, and 15.0°C. Subsequent to infection, all fish (spring and autumn conditions) were fed to satiation daily. Subsamples of juvenile herring were collected and frozen for chemical analysis immediately prior to the initiation of the experiment, on day 31 and at the completion of the experiment on day 52. Not all fish in the infected treatments contracted ichthyophoniasis, resulting in disproportionate sample sizes (Table 1). Lengths and weights of all fish were measured at experiment initiation and completion and for fish subsampled on day 31. Mortalities were removed from the experimental tanks daily and their lengths and weights were recorded. All samples and mortalities were assessed for infection via *in vitro* explant culture of the heart according to methods in Gregg et al. (this issue).

### 2.2. Chemical Analysis

Samples were analyzed for proximate composition including lipid, protein, moisture, and ash content according, standard procedures outlined in Vollenweider et al. [23] (Table 1). Fish selected for chemical analysis represented the entire size range of fish from a given treatment. Briefly, stomach contents were removed and whole fish were dried to a constant mass at 135°C in a LECO Thermogravimetric Analyzer (TGA) 601. Moisture content was calculated from wet and dry masses. Dried fish were homogenized and aliquots were randomly selected for each analysis. Lipid content was measured using a modification of Folch's method outlined by Christie [24] in a Dionex Accelerated Solvent Extractor with 2 : 1 (v : v) chloroform:methanol. Extracts were washed to remove coextractables and percent lipid was measured gravimetrically after solvent evaporated. Protein content was estimated from nitrogen content which was measured using a LECO Nitrogen Analyzer TruSpec following the Dumas method [25]. Protein samples were analyzed in duplicate to ensure accuracy. Ash content was measured gravimetrically after combusting samples at 600°C using the TGA. Quality assurance (QA) samples for each analysis were included with each batch of 17 samples, including blanks, reference material, and replicate sample. If QA samples fell outside prescribed limits, samples were reanalyzed. 

Energy content was calculated by summing the energy contributed by total-body lipid and protein as described in Vollenweider et al. [23]. Energy equivalents of 36.43 kJ g^−1^ for lipid and 20.10 kJ g^−1^ for protein were used [26].

### 2.3. Statistical Analysis

Allometries between energy and length were used to estimate the average energy content of fish in each of the treatment groups. Only a subset of fish from each treatment groups on days 0 and 52 were chemically analyzed. For each of these groups we constructed linear models relating the total energy content of fish to their lengths (*R* ^2^ > 0.50). The relationships were linearized by transforming the data to their natural logarithms before constructing the models. These resulting models were used to predict the energy content of each fish in a treatment group based on their lengths. Energy densities were calculated for each fish by dividing the predicted energy content by the observed fish weight. Subsequent analyses used the set of predicted values as the response variables for a given treatment. Analyses involving day 31 relied only on observed samples because no measurements of length or weight were recorded for the remaining fish. 

Total energy, energy density, and percent lipid were the response variables compared between treatments using a General Linear Model (GLM) for ANOVA and Tukey Test for multiple comparisons. For the first experiment, where fish were continuously fed, the model was

(1) $$\begin{array}{c} \text{Response}=\text{infection}+\text{time}+\text{infection}\times \text{time}. \end{array}$$

For the second experiment, where fish fasted and then infected upon refeeding, the model was

(2) $$\begin{array}{cc} & \text{Response}\\ & =\text{infection}+\text{time}+\text{temperature}+\text{infection}\times \text{time}\\ & \;+\text{temperature}\times \text{time}+\text{infection}\times \text{temperature}\times \text{time}. \end{array}$$

All factors were considered fixed for this study.

The rate of energy gain over the study period was calculated using a regression slope of natural log-transformed total energy content. Statistical differentiation of slopes was tested using a GLM ANOVA with transformed values of total energy content as the response, day ∗ an indicator variable as the model, and day and indicator variable as covariates following procedures outlined in Minitab 15 statistical package. Herring lengths were compared using 2-Sample *t*-tests and *T* statistics are provided. Statistical variation reported in the text and indicated in figures are standard errors.

A brief analysis of mortality was conducted. Nearly half (49%) of the mortalities occurred within the first five days of the experiment. Of these initial mortalities, only 21% were infected fish, suggestive of handling mortality. All subsequent mortality analysis excluded this data.

## 3. Results

### 3.1. Mortality Rates from Infection (Ambient Water Temperature)

Synergistic interactions of *Ichthyophonus* infection and fasting caused elevated mortality rates in juvenile herring beyond cumulative effects of the two treatments. “Spring herring” that had fasted prior to infection incurred the highest mortality rate over the course of the experiment (53%), followed by “autumn fish” that had been fed continuously prior to infection (37%). Control “spring herring” (uninfected) died at a similar rate as those that had been fed continuously (11% each), indicating that the fasting period was not long enough to cause irreparable nutritional stress (See Gregg et al. this issue, for a full discussion of mortality kinetics).

### 3.2. Energetic Cost of Infection in “Autumn Herring” (Ambient Water Temperature)

Compared to the uninfected controls, “autumn herring” with relatively good body condition incurred significant energetic costs from* Ichthyophonus* infection in ambient water temperatures. This was most apparent 31 days after infection, at which time the total energy content of infected juveniles declined by 58% from their initial condition while that of control fish increased by 32% (*F*_(1,38)_ = 0.54, *P* = .468) (Figure 2). Over the ensuing 21 days, infected fish recovered somewhat and stored energy at a rate 61% faster than controls (1520 versus 942 J d^−1^, *F*_(1,100)_ = 0.18, *P* = .677). Despite elevated energy storage, 52 days of refeeding did not result in infected fish acquiring significant energy since the time of infection (*F*_(1,41)_ < 0.01, *P* = .968). In contrast, controls gained appreciable energy over the same 52-day refeeding period at a rate of 698 J d^−1^, resulting in a net gain of 42% total energy (kJ) (*F*_(1,105)_ = 76.02, *P* < .001) and 22% energy density (kJ g^−1^) (*F*_(1,105)_ = 13.83, *P* < .001). Fifty-two days after infection, the energetic cost of infection in feeding juvenile herring relative to controls was a 30% reduction in total energy content (61.6 versus 87.4 kJ; *F*_(1,89)_ = 29.74, *P* < .001) and a 21% reduction in energy density (5.3 versus 6.7 kJ g^−1^, *F*_(1,89)_ = 10.12, *P* = .002).

Energetic costs of *Ichthyophonus* infection resulted in a depletion of lipid reserves. Despite continuous feeding, lipid content (% dry mass) of infected juvenile herring declined by 11% one month after infection (24.6 versus 22.0%), and remained similarly depleted by day 52 (22.6%; *F*_(1,13)_ = 0.71, *P* = .414) (Figure 2). In contrast, controls that had been fed continuously built up their lipid reserves by the same proportion (11%) after one month (24.6 versus 27.7%; *F*_(1,16)_ = 0.01, *P* > .924), and another 12% over the ensuing 21 days (27.7 versus 31.3%; *F*_(1,16)_ = 0.66, *P* = .428). Consequently, infected juvenile herring had 28% smaller lipid reserves than controls by the end of the 52-day study period (22.6 versus 31.3%; *F*_(1,13)_ = 1.84, *P* = .197).

### 3.3. Compensatory Response in “Spring Herring” (Ambient Water Temperature)

**Figure 3:**
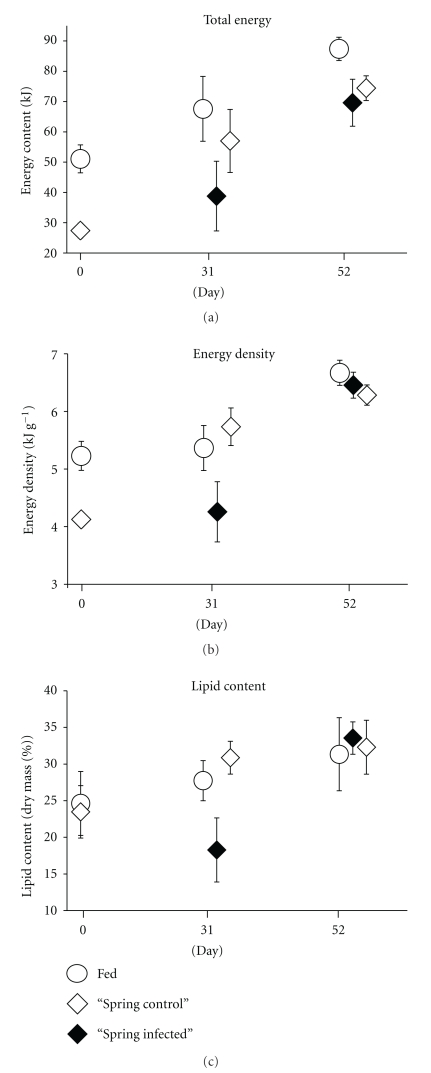
Compensatory response of young-of-the-year Pacific herring infected with *Ichthyophonus *depicted by total energy content, energy density, and lipid content (% dry mass). Fish represented in this figure were cultured in ambient water temperature (9.5°C). Low sample size of infected fish on day 31 precludes statistical tests.

*Ichthyophonus* infection delayed compensatory growth response following food deprivation in “spring herring”. To initiate a compensatory growth response, herring were fasted for 56 days to reduce their body condition to levels representative of wild fish in the spring following food-limited winter months. After the fasting period, total energy content of juvenile herring was 46% less than fed fish (27.4 ± 2.5 versus 51.1 ± 4.6 kJ; *F*_(1,56)_ = 12.23, *P* = .001), and energy densities were 21% less than fed fish (4.1 ± 0.1 versus 5.2 ± 0.3 kJ g^−1^; *F*_(4,56)_ = 4.69, *P* = .035) (Figure 3). During the first 31 days of refeeding controls demonstrated compensatory growth response, acquiring energy 79% faster than fish that had been fed continuously (954 versus 533 J d^−1^, *F*_(1,74)_ = 1.89, *P* = .173). Over the same refeeding period, infected fish did not exhibit compensatory response, gaining energy at a slower rate than continuously fed fish (367 versus 533 J d^−1^, *F*_(1,72)_ = 0.06, *P* = .813). As such, infected fish were in relatively poor condition compared to controls by day 31, with a 32% disadvantage in their total energy content (38.8 ± 11.5 versus 57.0 ± 10.4 kJ; *F*_(1,9)_ = 13.09, *P* = .006) and a 26% disadvantage in their energy density (4.3 ± 0.5 versus 5.7 ± 0.3 kJ g^−1^); *F*_(1,9)_ = 15.83, *P* = .003). 

Similar to “autumn herring”, the “spring herring” recovered from the initial impacts of infection to some degree during the second half of the experiment. Over the final 21 days of refeeding infected herring increased their rate of energy acquisition substantially which is indicative of compensatory growth response, gaining energy 49% faster than the continually fed fish (1400 versus 942 J d^−1^, *F*_(1,104)_ = 0.64, *P* = .425). Over the same refeeding period, controls no longer exhibited compensatory growth response, their energy acquisition rates moderating to that of continually fed fish (792 versus 942 J d^−1^, *F*_(1,163)_ = 0.14, *P* = .709). By the end of the 52-day experiment, infected herring were energetically on par with controls, only incurring a 6% reduction in total energy content relative to controls. (69.6 ± 7.8 versus 74.4 ± 4.1 kJ, resp.; *F*_(1,82)_ = 0.28, *P* = .596 and 6.5 ± 0.2 versus 6.3 ± 0.2 kJ g^−1^, resp.; *F*_(1,82)_ = 2.55, *P* = .114). Despite elevated energy gains, previously fasted fish never attained the same size as their continuously fed counterparts over the study duration. Hence the total energy of the previously fasted fish was lower than the continuously fed fish (73.6 ± 3.6 versus 87.4 ± 3.8 kJ, resp.; *F*_(1,160)_ = 4.57, *P* = .034) even though their energy densities were the same (6.5 ± 0.2 versus 6.3 ± 0.2 versus 6.7 ± 0.2 kJ g^−1^ for infected “spring herring”, control “spring herring,” and fed fish, resp.; *F*_(2,159)_ = 0.72, *P* = .488).

Lipid acquisition was also delayed following food deprivation. After the 52-day fasting period, lipid content (% dry mass) was reduced by 4% relative to that of herring that had been continually fed (23.5 ± 3.6 versus 24.6 ± 4.4% lipid; *F*_(1,14)_ < 0.01, *P* = .967) (Figure 3). One month after *Ichthyophonus* infection, lipid content declined significantly by an additional 22% despite refeeding (18.3 ± 4.4 versus 23.5 ± 3.6% lipid; *F*_(1,9)_ = 0.49, *P* = .504). Over the same time period, the compensatory response of control “spring herring” was so strong that their lipid content exceeded the lipid content of continually fed fish (30.9 ± 2.2 versus 25.2 ± 4.6; *F*_(2,14)_ = 7.71, *P* = .027). After 31 days, infected fish experienced a 41% lipid deficit relative to controls (18.3 ± 4.4 versus 30.9 ± 2.2% lipid; *F*_(1,9)_ = 17.90, *P* = .002). Over the following 21 days, infected herring recovered from their initial lipid deficit. Coincidentally, the accelerated rate of lipid storage in controls slowed. Thus by day 52, lipid content of all fish that had undergone previous food restrictions, regardless of infection, were equal to that of continually fed fish (33.6 ± 2.2 versus 32.3 ± 3.7 versus 31.3 ± 5.0% lipid, for infected “spring herring”, control “spring herring,” and fed fish, resp.; *F*_(2,20)_ = 17.90, *P* = .756).

### 3.4. Temperature Effects on “Spring Herring”

Water temperature influenced the lethality of *Ichthyophonus* infection on juvenile herring. Relative to control mortalities (uninfected fish), *Ichthyophonus*-induced mortalities were greatest in juvenile herring reared in ambient water temperatures (42% mortality), followed by hot (12%) and cold (4%) treatments. (See Gregg et al. this issue, for a complete discussion of mortality kinetics). 

**Figure 4:**
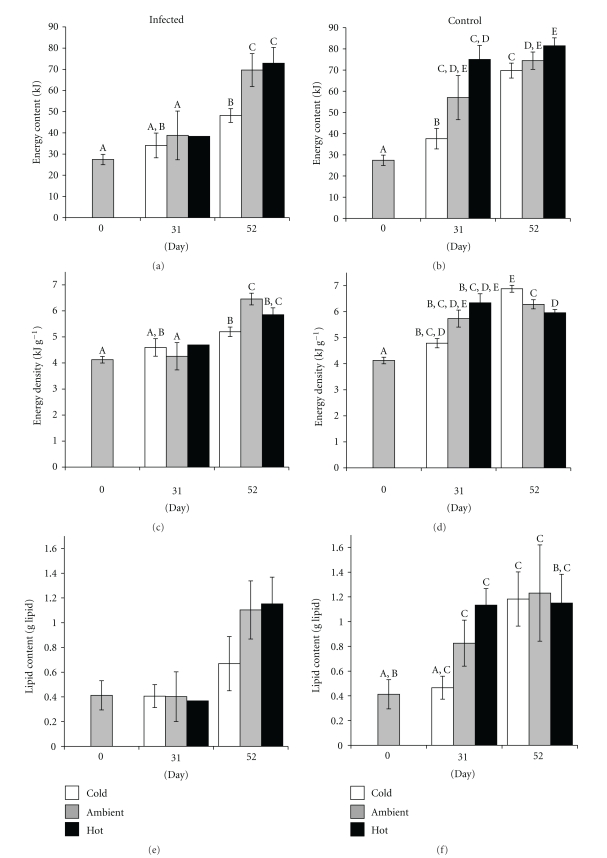
Temperature influence on the energetic cost of *Ichthyophonus *infection in “spring” young-of-the-year Pacific herring depicted by total energy content, energy density, and lipid content (g lipid). Water temperatures were cold (9.5°C), ambient (12.0°C), and hot (15.0°C). Different letters within a panel represent statistical differentiation. Lack of letters indicates no statistical differentiation. Low sample size of infected fish on day 31 precludes statistical tests.

Warmer temperatures conferred greater rates of energy acquisition in “spring herring” over the 52-day re-feeding period, a pattern which was more pronounced in infected fish. In the cold treatment, controls gained energy at a rate of 1167 J d^−1^ while those in ambient and hot treatments gained energy at rates 7% (1253 J d^−1^; *F*_(1,198)_ = 0.190, *P* = .662) and 18% (1424 J d^−1^; *F*_(1,227)_ = 1.390, *P* = .239) more rapidly, respectively (Figure 4). Temperature effects on energy storage rates were more pronounced in infected herring, which gained energy at rates 27% (1095 J d^−1^; *F*_(1,125)_ = 2.81, *P* = .096) and 37% (1265 J d^−1^; *F*_(1,121)_ = 5.19, *P* = .025) more rapidly in the ambient and hot treatments than in the cold treatment (797 J d^−1^). For a given temperature, energy storage rates of infected fish were depressed relative to controls. As temperature increased, the effect of infection on energy storage rates declined, with infected herring having disadvantages of 32%, 13%, and 11% in the cold (*F*_(1,170)_ = 6.47, *P* = .012), ambient (*F*_(1,153)_ = 0.55, *P* = .458) and hot temperatures (*F*_(1,178)_ = 0.39, *P* = .535), respectively. 

The influence of temperature and infection on energy storage rates was reflected in the total energy content of “spring herring”. After 31 days of refeeding, infected fish failed to gain energy in any water temperature (*F*_(6,107)_ = 10.47, *P* > .858). Only by day 52 had energy content increased significantly in all temperatures (*F*_(6,107)_ = 10.47, *P* < .001). In contrast, all controls had gained appreciable energy after 31 days (*F*_(6,271)_ = 86.49, *P* < .002) and continued to gain energy over the ensuing 21 days in the cold (*F*_(6,271)_ = 86.49, *P* < .001) and hot treatments (*F*_(6,271)_ = 86.49, *P* = .028). Within a given water temperature, infected fish always had reduced energy content relative to controls, though statistical differentiation was intermittent. After 31 days, energetic reductions in infected fish relative to controls were relatively large and increased with water temperature: 9%, 32% and 49% for cold (*F*_(1,9)_ = 0.29, *P* = .606), ambient (*F*_(1,9)_ = 13.09, *P* = .006) and hot (*F*_(1,9)_ = 2.94, *P* = 0.121) treatments, respectively. By day 52, energetic reductions of infected fish remained large, though the trend with increasing water temperature no longer held: 31%, 6%, and 11% for cold (*F*_(1,99)_ = 108.56, *P* < .001), ambient (*F*_(1,82)_ = 0.28, *P* = .596) and hot (*F*_(1,107)_ = .01, *P* = .927) treatments, respectively. 

**Figure 5:**
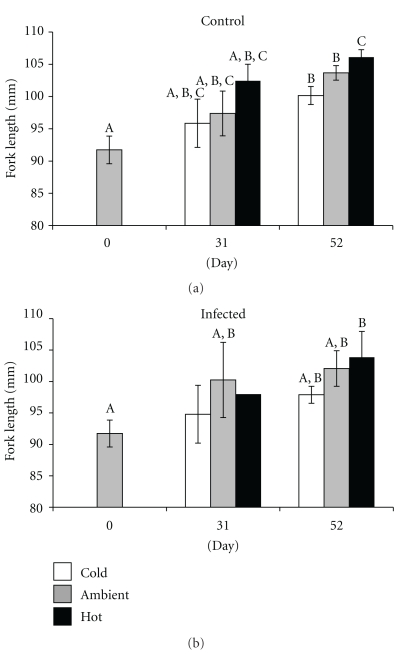
Growth rates of “spring” young-of-the-year herring over the course of the experiment under variable temperature conditions: cold (9.5°C), ambient (12.0°C), and hot (15.0°C). Different letters represent statistical differentiation. Lack of letters indicates no statistical differentiation. Low sample size of infected fish on day 31 precludes statistical tests.

Reduced total energy content of infected herring was partially attributed to decreased growth rates. For all but one day/temperature combination, infected herring were smaller than controls, though never statistically so (Figure 5, Table 2). Growth rates of juvenile herring increased as water temperature increased. Controls grew 0.16, 0.23, and 0.27 mm d^−1^ in cold, ambient, and hot temperatures, respectively, while infected herring grew 0.12, 0.20, 0.23 mm d^−1^ (Figure 5). *Ichthyophonus* infection had relatively little effect on growth rates. Among the controls, “spring herring” reared in the hot treatment grew 40% more rapidly than in the cold treatment and were significantly larger after 52 days (100.2 ± 1.4 versus 106.1 ± 1.2 mm; *T*(124) = −3.30, *P* = .001). Growth rates of infected fish were 48% greater in hot than cold treatments. Greater variation in the size of infected fish resulted in no statistical differences between mean fish sizes in different temperature treatments by day 52, though fish in the hot treatment were also nearly 6 mm larger on average than those in the cold treatment (97.9 ± 1.4 versus 103.9 ± 4.1 mm; *T*(15) = −1.38, *P* = .188).

Reduced somatic growth in the infected fish did not fully account for their lower total energy content, however, as energy densities were also lower in infected fish for each temperature (Figure 4, Table 2). Interaction effects of lipid content and temperature also contributed to decreased energy in infected herring. In the infected fish, lipid content remained depressed by day 31 in all temperatures and did not increase until day 52 (*F*_(6,33)_ = 1.85, *P* = .120). A similar delay in the recovery of lipid content was observed in the cold treatment for controls, though the delay did not occur in the warmer temperatures (*F*_(6,51)_ = 4.10, *P* = .002).

## 4. Discussion

### 4.1. Energetic Cost of Infection in “Autumn Herring” (Ambient Water Temperature)

Juvenile herring infected with* Ichthyophonus* incurred significant energetic costs, the magnitude of which depended on fish condition and consequently season at the time of infection. “Autumn herring” that fed continually and were in relatively good condition at the time of infection were representative of wild juvenile herring during peak energetic condition in the fall. The energy density of juvenile herring in our experiment at the time of infection (5.2 ± 0.3 kJ g^−1^) lies within peak seasonal values reported in November in Prince William Sound (4.9–7.5 kJ g^−1^) [21, 22]. Fifty-two days after infection, juvenile herring incurred a 20% reduction in energy density. To put this in perspective, the energetic cost of *Ichthyophonus* infection we measured over 52 days is similar to the energy density metabolized by fasting herring in a laboratory study of similar duration (23% loss from 5.2 kJ g^−1^over 55 days) [15]. Likewise, energetic costs incurred from *Ichthyophonus* infection are equal to 50–100% of the overwinter energy loss measured in the wild over approximately 120 days [15, 21, 27]. We conclude that herring infected with *Ichthyophonus* had no energy available for storage.

### 4.2. Compensatory Response in “Spring Herring” (Ambient Water Temperature)

Following food deprivation, *Ichthyophonus* infection caused an initial delay in the compensatory response of “spring herring”. Thirty-one days after infection and feeding commenced, lipid stores of infected fish continued to decline by an additional 22% despite *ad libitum* food supplies. This represents a 10% accelerated rate beyond the lipid catabolism associated with fasting alone. Coincidentally, control fish incurred a 38% increase in lipid content, demonstrating the initial cost of infection superseded compensatory response. Over the ensuing 21 days, however, the body composition of infected herring recovered to values similar for continually fed fish and “spring controls”, demonstrating herring's high capacity for compensatory response. Similar immediate reductions in lipid stores and energy content have been observed in juvenile halibut (*Hippoglossus stenolepis*) and channel catfish (*Ictalurus punctatus*). Initial declines in lipid and energy content facilitated rapid growth during the compensation phase [28, 29]. Similarly, infected herring had disproportionally greater growth rates than controls over the first 31 days post-infection. This was the only instance in which infected herring were larger than controls (Figure 5, day 31, ambient). As in the juvenile halibut and channel catfish [28, 29], lipid and energy content of infected herring was restored subsequently. This energetic strategy contrasts with other fish species such as juvenile Atlantic salmon (*Salmo salar*) and brown trout (*Salmo trutta*) which initially prioritize recovery of energy reserves and secondarily prioritize structural growth [30, 31]. 

The various energy allocation strategies suggest life history differences among species and maturation states. For example, initial restoration of energy and fat stores by juvenile Atlantic salmon may be a mechanism to enhance spawning [31] as suspended maturation has been demonstrated in males that fail to build sufficient energy reserves prior to the maturation process [32]. Juvenile herring likely elect to replenish energy and fat stores relatively quickly in order to provide endogenous energy for winter when prey is scarce. Energy stores in the autumn have been identified as a major factor in their overwinter survival [21, 22].

The capacity to rapidly recover energetic condition and size during a compensatory phase following food or growth restrictions does not come without long-term costs. Of greatest concern are potential reductions in immune capacity [33]. By spring, herring have undergone several months of severe food restrictions. Spring plankton blooms likely stimulate a compensatory response. Coincidentally, excluding periods of epidemics, *Ichthyophonus* prevalence in herring is generally greatest in spring, albeit in spawning schools [1, 8]. If spawning schools and juvenile nursery areas overlap spatially, spring may be the time in which herring are most susceptible to infection due to the combined effects of (1) reduced energy state from fasting and (2) decreased immune capacity from compensatory growth.

Other deleterious effects of compensatory growth include prolonged periods of poor growth and condition despite initial restoration of lipid reserves [31]. Poor growth may result in reduced swimming capacity [34, 35]. *Ichthyophonus* infection alone can also reduce swimming performance [11], thus there is potential for additive or synergistic effects of infection and compensatory response on swimming performance, especially as water temperatures increase [12]. Reduced swimming capacity could lead to increased vulnerability to predation [36] or declines in foraging efficiency and thus starvation. Some fish species have demonstrated the capacity to suppress metabolism under conditions of food deprivation, however, which could mediate costs associated with compensatory growth [37].

### 4.3. Temperature Effects on “Spring Herring”

Over the temperature range we examined (9.5–15.0°C), temperature had a significant impact on the energetic costs of* Ichthyophonus* infection in “spring herring”. Increasing water temperatures accommodated greater lipid and energy acquisition in juvenile herring. As a result, we observed the greatest impact of infection in cold temperatures in which energy acquisition rates were lowest. Water temperature ranges used in this study were typical of Puget Sound, however, they encompassed the optimal water temperature for juvenile Pacific herring growth of 12.2°C [38]. Alaskan water temperatures are cooler than those of Puget Sound, seasonally ranging from 4.5–10°C at 25 m water depth in Prince William Sound [22] and 6.5–13.5°C in southeast Alaska [39]. Thus the impacts of *Ichthyophonus* may be of greater consequence in colder Alaskan waters.

*Ichthyophonus* infection confers significant energetic costs to juvenile herring, though the mechanism behind the costs is unknown. Reduced consumption rates of infected fish likely contribute to their decreased body condition. Though consumption was not directly measured in this experiment, observational evidence suggests that infection did reduce feeding rates. Temperature-dependent consumption rates could additionally confound differences in body condition by infection and temperature. However, energetic costs incurred by infected herring were greater than would otherwise be incurred by starvation alone, suggesting additional costs likely associated with immune function are at play.

### 4.4. Summary

We found differential energetic impacts imposed by *Icthyophonus* infection depending on herring's condition at the time of infection. Among “autumn fish” feeding *ad libitum* with good body condition, infection caused a 30% decrease in total energy content after 52 days which is comparable in magnitude to overwinter losses in the wild which occur in less than half the time. Among “spring herring” having undergone extensive fasting with poor body condition, the energetic toll of *Ichthyophonus* infection initially delayed recovery of body composition. One month after re-feeding infected fish showed no sign of compensatory response and their total energy content was reduced by 32% relative to controls. During the subsequent 21 days compensatory response became evident, however, and the energetic costs of infection were significantly reduced such that infected fish only had a 6% reduction in total energy content relative to controls. Despite initial delays, compensatory response appears to mediate energetic costs of infection eventually, though long term consequences are unknown. Energetic impacts of infection were greatest in colder temperatures, suggesting *Ichthyophonus*-induced reductions in body condition may have greater consequences in the northern extent of herring's range where juveniles use most of their energy reserves to survive their first winter.

## Figures and Tables

**Table 1:** Sample sizes of juvenile Pacific herring for proximate composition analysis by temperature, where Infect = infected and Control = controls.

Feeding history	Fed-“Autumn”		Fasted-“spring”					
Temperature	Ambient		Cold		Ambient		Hot	
Sampling Day	Infect	Control	Infect	Control	Infect	Control	Infect	Control
0		8			8	8		
31	1	11	5	7	4	8	1	9
52	8	8	8	8	8	8	7	11

**Table 2:** Test statistics for comparisons of infected versus uninfected juvenile Pacific herring lengths and energy densities at different water temperatures. Length comparisons were 2-sample *t*-tests (*T* statistic reported), while energy density comparisons were GLM ANOVAs (*F* statistic reported).

	Cold			Ambient			Hot		
	*P*-value	statistic	df	*P*-value	statistic	df	*P*-value	statistic	df
*Length*									

Day 31	.863	0.18	8	.695	−0.42	5	.604	0.53	15
Day 52	.245	1.17	99	.605	0.53	18	.604	0.53	15

*Energy Density*									

Day 31	.552	0.38	1,9	.003	15.83	1,9	.246	1.54	1,9
Day 52	<.001	69.66	1,99	.114	2.55	1,82	.708	0.14	1,107
